# Mechanical Properties and Constitutive Model of Rapid-Curing Epoxy Resin Concrete Under Different Temperature Conditions

**DOI:** 10.3390/ma19050996

**Published:** 2026-03-05

**Authors:** Nannan Sun, Chuandong Shen, Jingwen Shen, Yuzhu Wang

**Affiliations:** 1 School of Materials and Architectural Engineering, Guizhou Normal University, Guiyang 550025, China; 2017021013@chd.edu.cn; 2 The Third Construction Engineering Company Ltd. of China Construction Second Engineering Bureau, Beijing 100070, China; shenchuandong1991@126.com; 3 Yantai Research Institute, Harbin Engineering University, Yantai 264000, China; sjw@hrbeu.edu.cn

**Keywords:** epoxy resin concrete, rapid curing, temperature sensitivity, mechanical properties, constitutive model

## Abstract

Recently, epoxy resin concrete (ERC) has shown significant potential in rapid repair applications, such as bridge expansion joints, owing to its early strength gain, rapid hardening, excellent adhesion, and durability. Based on the background of rapid repair scenarios for small- and medium-span bridges, this study designed a mix proportion of ERC. A systematic investigation was conducted on its mechanical properties and constitutive model under various curing temperatures (5 °C, 20 °C, and 35 °C) and ages. Experimental results indicate that the designed ERC cures within 2 to 6 h and achieves a compressive strength of 15 MPa at 1 day, meeting the requirement for early traffic reopening. Both material strength and elastic modulus increase significantly with age, reaching a compressive elastic modulus of 16 GPa at 90 days. Based on the measured uniaxial compressive and tensile stress–strain data, a temperature-dependent constitutive model was established. The fitting parameters exhibit a quadratic functional relationship with curing temperature. The model demonstrates high fitting accuracy under all tested conditions (*R*^2^ ≥ 0.9293). This study provides a theoretical basis and data support for the application and numerical simulation of ERC in bridge engineering.

## 1. Introduction

The prolonged service life of transportation infrastructure has accentuated the need for effective maintenance and repair of highway bridges [1,2,3]. Critical elements such as bridge expansion joints are particularly susceptible to cracking, damage, and displacement due to cyclic traffic loads and environmental exposure, compromising structural durability and safety [4,5,6]. Repairs in these areas require materials that cure rapidly and develop early strength to enable quick traffic reopening, thereby minimizing socio-economic disruptions [7]. Consequently, there is significant engineering value in developing rapidly curing, high-early-strength repair materials that are adaptable to varying climatic conditions and easy to place [8,9].

Epoxy resin concrete (ERC), as a high-performance polymer-based composite material, utilizes epoxy resin as a binder combined with properly graded fine aggregates. It cures rapidly at room temperature, achieves high early strength and exhibits excellent bonding capability, mechanical properties, and durability. These characteristics make it particularly suitable for rapid repair and performance restoration of structural components such as bridge deck pavement, connection plates, and expansion joints [10,11,12,13].

In recent years, extensive research has been conducted on ERC, focusing on mix proportion design, mechanical properties, and repair applications [14,15]. Studies have optimized mix proportions by examining the effects of resin-to-filler and matrix-to-aggregate ratios on performance [16]. Incorporating epoxy resin into cementitious grouts enhances long-term compressive strength, tensile strength, modulus of elasticity, toughness, and chemical resistance, despite prolonging curing time [17]. Furthermore, research has identified that testing conditions—including specimen geometry, loading rate, and temperature—significantly influence tensile properties [18]. The effects of resin content and fiber reinforcement on strength and workability have also been systematically investigated [19]. Additionally, ERC has been evaluated in specialized applications, such as epoxy-modified porous concrete for pavements, where it improves mechanical performance and extends service life by reducing tensile stresses [20].

Despite its application in engineering projects, fundamental research on ERC remains underdeveloped. Key limitations include: (1) insufficient systematic evaluation of rapid-curing ERC, particularly regarding early-age mechanical evolution and workable time windows during construction; (2) inadequate investigation of strength development and deformation under varying curing temperatures, which restricts its applicability across diverse climatic zones; and (3) the absence of a unified constitutive model suitable for finite element simulations, limiting deeper structural analysis and design.

Addressing these gaps, this study systematically investigates the temperature-dependent constitutive modeling of ERC for the rapid repair of bridge expansion joints. An optimal mix proportion was first developed to achieve rapid curing. Comprehensive mechanical tests were then conducted at three curing temperatures (5 °C, 20 °C and 35 °C) and multiple ages to analyze performance evolution and temperature sensitivity. Finally, based on the experimental stress–strain relationships, a constitutive model was established, with its parameters explicitly defined as quadratic functions of curing temperature. This work bridges a critical research gap by providing both experimental data and a constitutive model for ERC behavior under varying thermal conditions, thereby supporting its reliable application and numerical simulation in diverse climatic environments.

## 2. Materials and Experiments Methods

This study aims to develop an ERC suitable for the rapid repair of bridge expansion joints, and systematically evaluate its mechanical properties and deformation characteristics under different curing temperatures. A comprehensive experimental framework was established, covering mix ratio design, specimen preparation, testing methods, and procedures.

**Figure 1:**
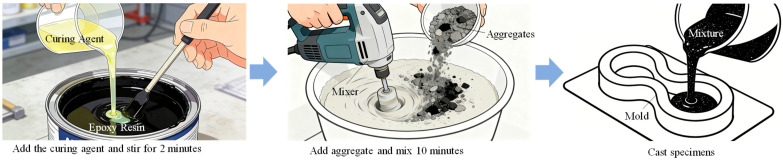
Schematic diagram of ERC specimen preparation.

(1) An optimal mix proportion for ERC was determined by screening 22 preliminary formulations based on setting time and early strength. The selected proportion consists of 450 kg of black epoxy resin, 74 kg of light-yellow curing agent, and 1950 kg of graded crushed stone aggregate per cubic meter of material. All subsequent experimental work is based on this optimized mix proportion.
(2) The mixing and specimen preparation process is illustrated in Figure 1. After precise weighing of the components, the epoxy resin and curing agent were initially blended in a mixing container for 2 min, followed by 10 min of mixing with aggregates to achieve homogeneity. Prior to casting, the molds were coated with a release agent. Specimens were consolidated using a vibrating rod or table. Due to the material’s self-compacting nature and short curing time, they were cured in molds under controlled temperature until testing.

(3) To account for climatic variations across China and the temperature sensitivity of ERC, three curing conditions were adopted: 5 °C (low temperature), 20 °C (room temperature), and 35 °C (high temperature). These simulate typical on-site temperatures during bridge maintenance. Mechanical tests—including compressive strength, tensile strength, and compressive elastic modulus—were performed on specimens cured at each temperature. Testing ages ranged from ultra-early stages (4 h, 8 h) to long-term periods (90 d, 180 d) to fully characterize property evolution and temperature dependence.
(4) The specimen types, dimensions, quantities and corresponding testing standards are detailed in Table 1. Testing strictly followed current national and industry standards to ensure the scientific validity and comparability of the results. In accordance with BS 6319 [21], which provides test methods for epoxy resin-based composites, the relevant evaluations in this study were conducted. Compressive strength was measured on 100 mm cubes, and tensile strength was determined using “8”-shaped specimens, both in accordance with British Standards [21]. Since BS 6319 [21] does not provide a method for elastic modulus, its measurement followed JTG E30-2005 [22], using 40 mm × 40 mm × 160 mm prismatic specimens.

(5) To ensure loading accuracy and test data repeatability, a constant loading rate was uniformly applied across all tests to avoid non-structural errors caused by fluctuations during the loading process.

## 3. Experimental Results

### 3.1. Compressive Strength and Failure Modes

**Figure 2:**
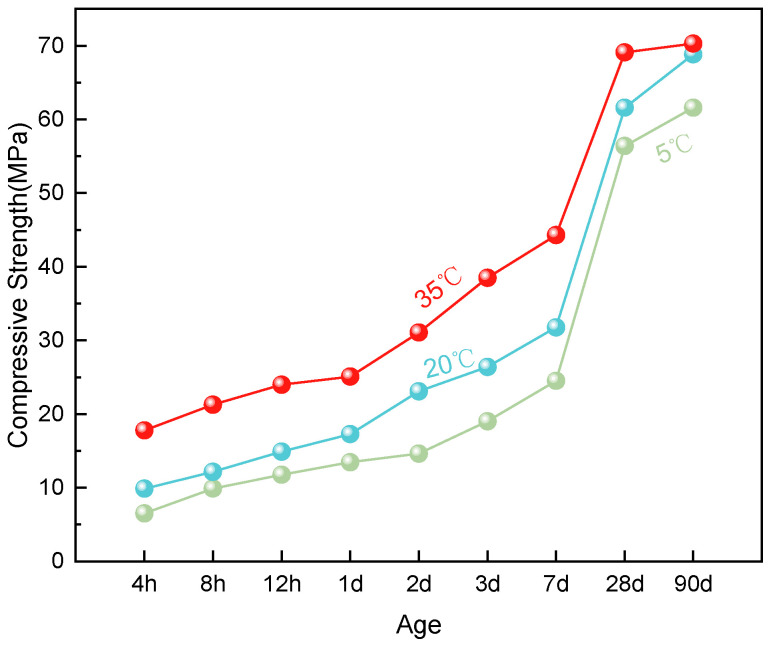
Compressive strength of ERC.

Compressive strength tests were conducted on specimens cured at three temperatures (5 °C, 20 °C, and 35 °C) and tested at ages ranging from 4 h to 90 days. The test results are illustrated in Figure 2.

The test results indicate that compressive strength correlates positively with curing temperature. The 35 °C group exhibited the fastest strength gain, reaching 15.9 MPa at 4 h, followed by the 20 °C and 5 °C groups. The most pronounced strength differences occurred during the initial curing period (4 h to 3 days), highlighting the strong accelerating effect of higher temperatures on early strength development. Strength evolution follows a biphasic trend: rapid growth up to 28 days, followed by a slower phase thereafter, with a notable inflection near 28 days. For instance, at 28 days the compressive strength already exceeded 90% of the 90-day strength at 20 °C. By 90 days, strength growth slowed and differences between temperature groups diminished. Although early-age strength under low-temperature curing (5 °C) was notably delayed—reaching only about 60% of the 20 °C value—it gradually increased in later stages, eventually approaching the levels observed at 20 °C. This phenomenon suggests that while temperature accelerates early development, ultimate strength is governed primarily by material composition.

Furthermore, the failure modes of specimens varied significantly with curing temperature, as summarized in Table 2. Overall, the compressive failure mechanism of ERC differs distinctly from the brittle crushing typical of ordinary cement concrete. The absence of coarse aggregates and the superior bonding provided by the epoxy matrix enable ERC to delay crack initiation and propagation under compression. This results in a dense internal network of fine cracks, while external cracking remains limited, preserving specimen integrity without significant surface spalling.

As shown in Table 2, during the early stage, the low-temperature specimens typically exhibited penetrating vertical cracks, showing typical brittle failure characteristics. In contrast, specimens cured at high temperature displayed a combined failure mode involving bulging and longitudinal cracking, indicating a certain tendency toward plastic failure. Beyond 28 days, the 5 °C specimens continued to exhibit higher deformation, resembling behavior of 20 °C and 35 °C specimens at the early stage. Meanwhile, specimens cured at 20 °C and 35 °C transitioned to brittle failure dominated by vertical cracking and surface spalling.

This evolution is attributed to ERC’s initially low elastic modulus and high deformability, which impart early plasticity. With age, its modulus increases toward that of cement concrete, deformability declines, and the material shifts from plastic to brittle behavior. This suggests that temperature not only influences the rate of strength development but also significantly modulates the failure mechanism and early-age deformation capacity of ERC.

### 3.2. Tensile Strength and Fracture Modes

**Figure 3:**
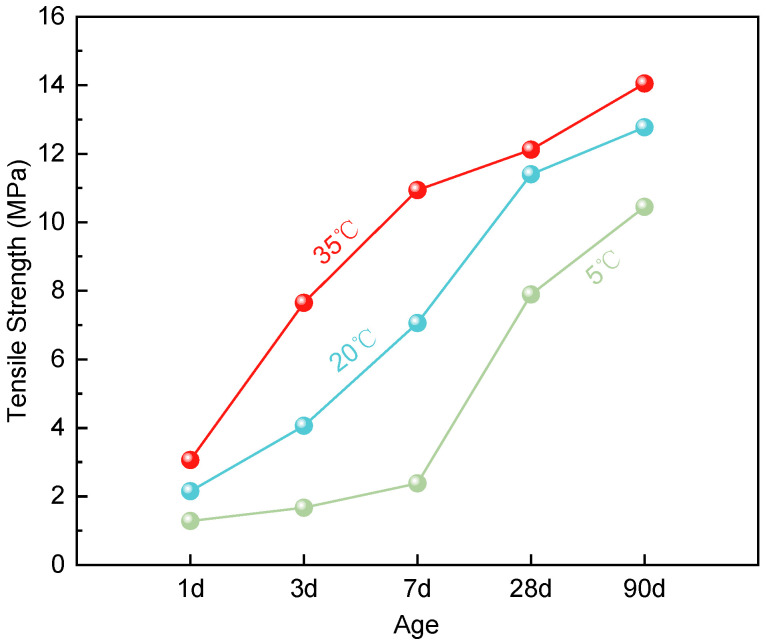
Tensile strength of ERC.

The tensile results of ERC are presented in Figure 3. Tensile strength generally increases with both curing age and temperature. At 35 °C, strength reaches approximately 78% of its ultimate value by 7 days, while at 20 °C it stabilizes around 28 days. In contrast, growth is notably slower at 5 °C, with the final tensile strength approximately 18% lower than that at 20 °C.

Similar to the compressive strength results, tensile strength increases with curing temperature. The 35 °C group exhibited the fastest gain, reaching 14.05 MPa at 90 days, compared to 10.45 MPa for the 5 °C group. The most pronounced differences occurred during early curing (1–7 days). For example, at 7 days the 35 °C group attained 10.94 MPa, whereas the 5 °C group achieved only 2.38 MPa. All temperature groups experienced a rapid increase phase from 1 to 28 days, after which growth decelerated. Unlike compressive strength, tensile strength differences between temperature groups remained evident even at 90 days. This suggests that higher temperatures not only promote early strength but also enhance long-term tensile capacity.

Table 3 summarizes the tensile failure modes across different ages and curing temperatures, which can be categorized into three types: (1) specimens with fine cracking but no complete fracture; (2) specimens with irregular fracture surfaces exposing aggregates; and (3) specimens with smooth, flat fracture interfaces.

At low temperature (5 °C) before 7 days, failure mainly involved cracking at the cross-section transition zone. Strong epoxy bonding allowed slow development of dense, ductile cracks. The testing machine stopped upon detecting crack initiation in the specimen, thus preventing complete fracture. By 28 days under low-temperature, failure still occurred at the transition zone but with aggregate exposure, indicating that the softer resin could no longer fully bind the aggregates. A similar pattern was observed in room-temperature specimens before 7 days.

In contrast, room-temperature specimens at 28 days and 90 days, as well as all high-temperature (35 °C) specimens, predominantly exhibited brittle fracture with regular, smooth surfaces. Higher temperatures and longer aging produced increasingly flat fractures. Thus, ERC tensile behavior shifts from an asphalt-like viscous-ductile response in the early stage to a cement-like brittle failure at later ages.

**Figure 4:**
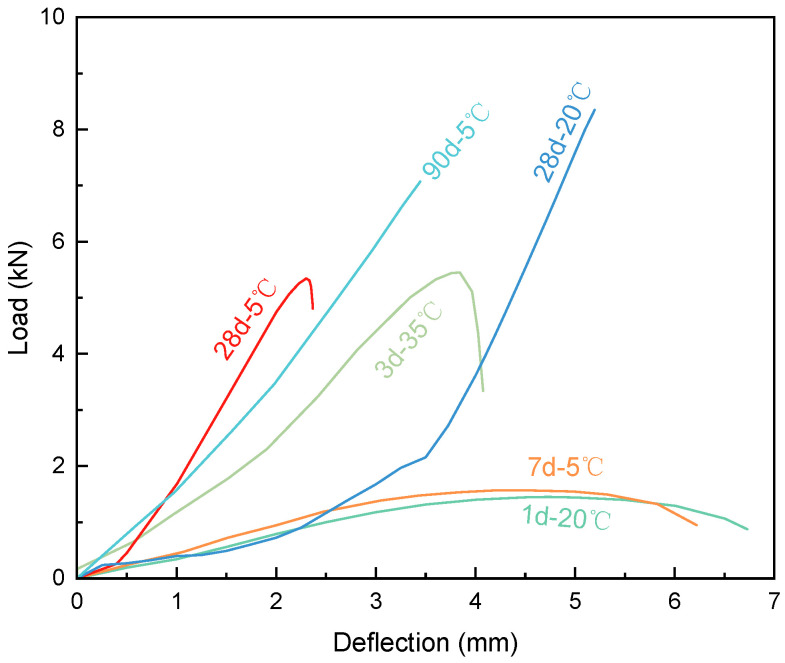
Typical load–displacement curves of ERC under tension.

Their load–displacement curves (Figure 4) reflect that specimens under early age or low-temperature conditions exhibit greater deformation and lower peak loads. The gradual post-peak descending branch indicates that ERC possesses a certain degree of plastic deformation capacity. With higher temperatures or longer ages, the curves become steeper and display a post-peak drop, corresponding to uneven fracture surfaces. After 28 days, the curves rise steeply and break abruptly at peak load without a descending branch, indicating fully brittle fracture.

In summary, both temperature and age strongly govern the tensile failure behavior of ERC. Low temperatures and early ages promote ductile, multi-crack failure with gradual post-peak softening, while higher temperatures and extended aging drive a transition to brittle fracture characterized by smooth surfaces and abrupt load loss.

### 3.3. Compressive Elastic Modulus

**Figure 5:**
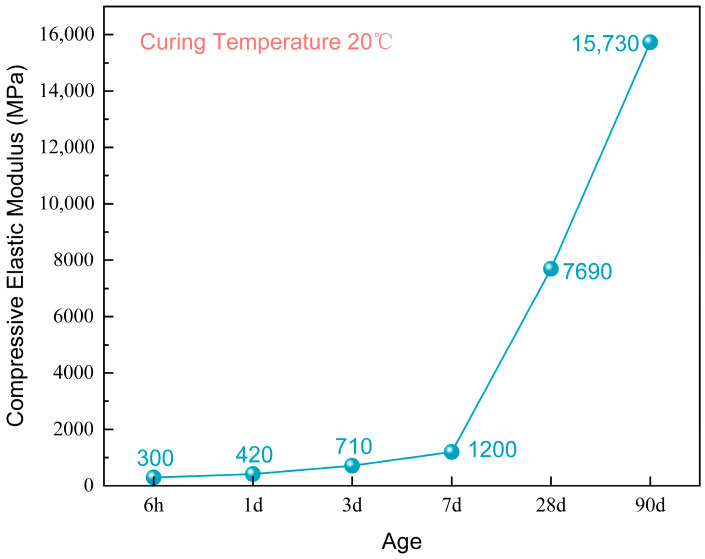
Compressive elastic modulus of ERC.

The compressive elastic modulus of ERC is shown in Figure 5. As the curing age increased, the elastic modulus rose rapidly from an early-age value of approximately 300 MPa to 15.7 GPa at 90 days, representing an increase of more than 50 times.

After 7 days of curing, the growth rate of the elastic modulus accelerated markedly, reflecting the stiffness leap characteristic of the material during its mid- to late-stage structural hardening phase. Compared to normal cement concrete, ERC exhibits a significantly lower modulus at early ages, providing greater strain tolerance. This property is beneficial for stress relief and crack mitigation in bridge expansion joints during the initial service period. In the long term, however, the stiffness of ERC gradually approaches that of normal cement concrete, contributing to the dual requirements of sustained load-bearing capacity and structural compatibility in deformation.

## 4. Constitutive Model of the Uniaxial Stress–Strain Behavior of ERC

### 4.1. Background and Theoretical Basis for the Constitutive Model

For the refined design and numerical simulation of critical ERC components, it is essential to establish a constitutive model capable of accurately describing its stress–strain response.

Numerous studies have shown that the stress–strain curves of various types of concrete under uniaxial compression and tension exhibit a certain consistency in form [23,24,25,26,27,28]. By adopting normalized dimensionless processing and introducing mathematical expressions, the universality, adjustability, and engineering applicability of the model can be significantly enhanced [29]. Existing curve-fitting approaches commonly employ mathematical forms such as polynomials, exponential functions, trigonometric functions, and rational fractions. Among these, the ascending branch is often fitted using cubic polynomials to ensure curve continuity and elastic transition, while the descending branch tends to be modeled using rational fractions to accurately capture the steep drop characteristic of brittle failure behavior [29].

Based on the theoretical framework of the typical concrete stress–strain curve proposed by Zhenhai Guo [30,31,32], this paper establishes uniaxial compressive and tensile constitutive models capable of characterizing temperature sensitivity. The models mathematically satisfy the typical eight geometric feature constraints, and through regression analysis, functional relationships between the model parameters and temperature were established to achieve unified predictive capability under multiple working conditions.

The constitutive model parameters were derived from the normalized stress–strain curves of ERC under uniaxial compression or tension. The procedure included: (1) normalizing experimental curves by peak stress and corresponding strain; (2) fitting key characteristic points of the curve (such as the slope of the elastic segment, peak point, and shape parameters of the descending branch) using the least squares method; and (3) establishing quantitative relationships between parameters and curing temperature through regression analysis.

### 4.2. Uniaxial Compressive Stress–Strain Constitutive Model

#### 4.2.1. Compression Test Results and Characteristic Analysis

**Figure 6:**
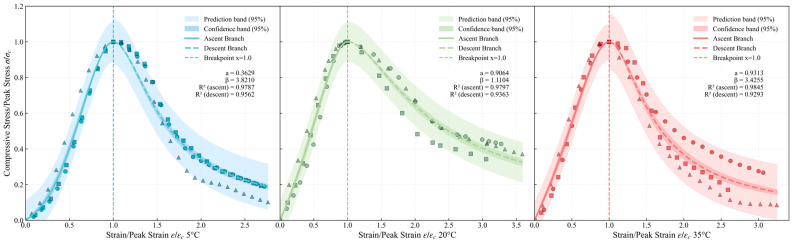
Full-curve model of ERC under uniaxial compression.

The load–displacement data from uniaxial compression tests at 28 days under 5 °C, 20 °C, and 35 °C were recorded by the testing machine and converted into stress–strain relationships. The data points were then normalized by defining the horizontal coordinate as the ratio of strain to peak strain and the vertical coordinate as the ratio of compressive stress to peak stress. The processed experimental results are presented as three different shapes of point sets in Figure 6. The results indicate that the overall trend of the ERC compression curves is similar to that of conventional cement concrete, exhibiting a nonlinear evolution characterized by a gradual initial ascent, followed by a sharp decline after reaching the peak, which reflects distinct brittle failure behavior.

#### 4.2.2. Compression Model Form and Parameter Definition

Based on the aforementioned curve characteristics and concrete constitutive model presented by Zhenhai Guo [30,31,32], a uniaxial compressive constitutive model for ERC is proposed, which includes two parts: the ascending branch and descending branch.

From the ascending branch ($\varepsilon /\varepsilon_{c}\leq 1$), a cubic polynomial is adapted, as shown in Equation (1), to ensure the continuity and differentiability of the curve at both the origin and the peak point.

(1) $$\frac{\sigma }{\sigma_{c}}=a\frac{\varepsilon }{\varepsilon_{c}}+(4-3a){\left(\frac{\varepsilon }{\varepsilon_{c}}\right)}^{3}+(2a-3){\left(\frac{\varepsilon }{\varepsilon_{c}}\right)}^{4}$$

In the equation, *σ* represents stress and *ε* represents strain. *σ_c_* is the peak stress and *ε_c_* is the peak strain. The parameter *a* is a dimensionless shape control parameter, defined as the ratio of the initial tangential modulus to the peak secant modulus. Its value directly influences the initial stiffness and the curvature of the ascending branch of the stress–strain curve.

Similarly, for the descending branch ($\varepsilon /\varepsilon_{c}>1$), a rational fraction is formulated, as shown in Equation (2), which effectively captures the rapid stress decay observed in the post-peak stage:

(2) $$\frac{\sigma }{\sigma_{c}}=\frac{\frac{\varepsilon }{\varepsilon_{c}}}{\beta {\left(\frac{\varepsilon }{\varepsilon_{c}}-1\right)}^{2}+\frac{\varepsilon }{\varepsilon_{c}}}$$

where *β* is the fitting parameter that controls the slope and curvature of the descending section.

As shown in Figure 6, which presents the fitted full curves of ERC under three temperatures, the modified compression full-curve model proposed in this study covers almost all data points of the experimental curves and fully captures the complete uniaxial compressive stress–strain response of ERC.

As can be seen from Figure 6, curing temperature significantly influences the shape of the stress–strain curves. At 5 °C, the narrow 95% confidence bands for both the ascending and descending branches indicate low data scatter and stable mechanical behavior. At 20 °C and 35 °C, the ascending branch shows a narrow confidence band and a high goodness of fit, while the descending branch exhibits a wider confidence band and increased data scatter, suggesting greater uncertainty in post-peak softening behavior.

For all temperatures, the *R*^2^ values for the ascending branch exceed 0.97, indicating that the model fits the elastic/plastic hardening stage exceptionally well. The *R*^2^ values for the descending branch range between 0.9293 and 0.9562, which are slightly lower than those for the ascending branch. This is because the mechanical behavior during the softening stage is more complex and is influenced to a greater extent by factors such as micro-crack propagation.

#### 4.2.3. Compression Temperature Sensitivity and Unified Expression

Assuming that parameters *a* and *β* exhibit significant temperature dependence, both can be described by quadratic functions of temperature *T*:

(3) $$a(T)=A_{1}+B_{1}T+C_{1}T^{2}$$

(4) $$\beta (T)=A_{2}+B_{2}T+C_{2}T^{2}$$

where *T* is the temperature, and *A*_1_, *B*_1_, *C*_1_, *A_2_, B*_2_, *C*_2_ are the correlation coefficients of the quadratic functions.

Substituting Equations (3) and (4) into Equations (1) and (2) constructs a unified compressive constitutive model for ERC, as shown in Equation (5):

(5) $$\frac{\sigma }{\sigma_{c}}=\left\{\begin{array}{l} a(T)\frac{\varepsilon }{\varepsilon_{c}}+[4-3a(T)]{\left(\frac{\varepsilon }{\varepsilon_{c}}\right)}^{3}+[2a(T)-3]{\left(\frac{\varepsilon }{\varepsilon_{c}}\right)}^{4},\varepsilon /\varepsilon_{c}\leq 1\\ \frac{\frac{\varepsilon }{\varepsilon_{c}}}{\beta (T){\left(\frac{\varepsilon }{\varepsilon_{c}}-1\right)}^{2}+\frac{\varepsilon }{\varepsilon_{c}}},\;\;\;\;\;\;\;\;\;\;\;\;\;\;\;\;\;\;\;\;\;\;\;\;\;\;\;\;\;\;\;\;\;\;\;\;\;\;\;\;\;\;\;\;\;\;\;\varepsilon /\varepsilon_{c}>1 \end{array}\right.$$

By applying quadratic function fitting to the shape control parameters *a* and *β* obtained from the regression analysis in Figure 6, the temperature-dependent quadratic correlation coefficients were derived, with the results presented in Table 4. The goodness of fit was *R*^2^ = 1.0 for the ascending branch and *R*^2^ = 1.0 for the descending branch, respectively, indicating that the model possesses good predictive ability.

### 4.3. Uniaxial Tensile Stress–Strain Constitutive Model

#### 4.3.1. Tension Test Results and Characteristic Analysis

**Figure 7:**
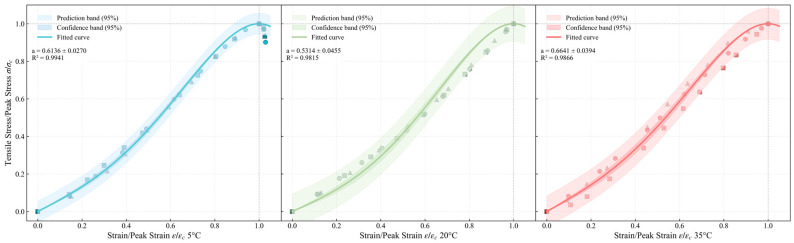
Full curve model of ERC under uniaxial tension.

Similar to the data processing method used for the compressive strength test, the tensile experimental results are shown in Figure 7 with three different shapes of point sets. The results indicate that the tensile curve exhibits a significant linear ascending trend up to the peak. However, at 20 °C and 35 °C, the specimens failed in a brittle fracture, and the testing system was unable to capture a reliable post-peak descending response. Therefore, the tensile constitutive model developed in this study considers only the ascending branch of the stress–strain curve.

#### 4.3.2. Tension Model Form and Parameter Definition

Given the absence of a distinct post-peak softening stage in the tensile response, the constitutive model focuses on fitting the ascending branch. A fifth-order polynomial is adopted for the fitting and is expressed as follows:

(6) $$\frac{\sigma }{\sigma_{c}}=a\frac{\varepsilon }{\varepsilon_{c}}+(2.5-2a){\left(\frac{\varepsilon }{\varepsilon_{c}}\right)}^{3}+(a-1.5){\left(\frac{\varepsilon }{\varepsilon_{c}}\right)}^{5},\;\varepsilon /\varepsilon_{c}\leq 1$$

where *σ* is the tensile stress, *σ_c_* is the corresponding peak stress, *ε* is the strain and *ε_c_* is the peak strain. The parameter *a* is the dimensionless shape control parameter for the ascending branch. The fitting results indicate that parameter *a* also follows a quadratic variation with temperature *T*.

As shown in Figure 7, which presents the fitted tensile stress–normalized strain relationship of ERC at three temperatures (5 °C, 20 °C, and 35 °C), the mechanical behavior under different temperatures is clearly illustrated through fitted curves, prediction bands, and confidence bands.

Across all temperatures, the ascending branch achieves *R*^2^ > 0.97, indicating an excellent fit with high agreement between experimental data and proposed constitutive model. At 5 °C, the confidence and prediction bands are the narrowest, with data points closely distributed around the fitted curve, suggesting the most stable and reproducible tensile behavior at low temperature. The confidence and prediction bands at 20 °C are the widest, indicating the greatest variability in mechanical behavior at room temperature.

#### 4.3.3. Tension Temperature Sensitivity and Unified Expression

Similar to the compressive constitutive model, assuming that parameter *a* exhibits significant temperature dependence, it can be described by a quadratic function of temperature *T*:

(7) $$a(T)=A+BT+CT^{2}$$

where *T* is the temperature and *A*, *B*, and *C* are the correlation coefficients of the quadratic function. Finally, the unified tensile constitutive model is formulated as:

(8) $$\frac{\sigma }{\sigma_{c}}=a(T)\frac{\varepsilon }{\varepsilon_{c}}+[2.5-2a(T)]{\left(\frac{\varepsilon }{\varepsilon_{c}}\right)}^{3}+[a(T)-1.5]{\left(\frac{\varepsilon }{\varepsilon_{c}}\right)}^{5},\;\varepsilon /\varepsilon_{c}\leq 1$$

The fitting results for the quadratic temperature-dependent coefficients are summarized in Table 5. The coefficient of determination *R*^2^ reaches 1.0, demonstrating that the model also exhibits excellent fitting and predictive capability under tensile loading.

## 5. Discussion

### 5.1. Mechanistic Diagram

Based on systematic experimental data and modeling analysis, this study investigated the material adaptability, performance evolution mechanisms, and constitutive model applicability of ERC in the rapid repair of bridge expansion joints. The analysis focuses on how curing temperature and age affect mechanical behavior, clarifying the temperature-dependent response of ERC and its comparative advantages and limitations relative to other epoxy-based materials.

**Figure 8:**
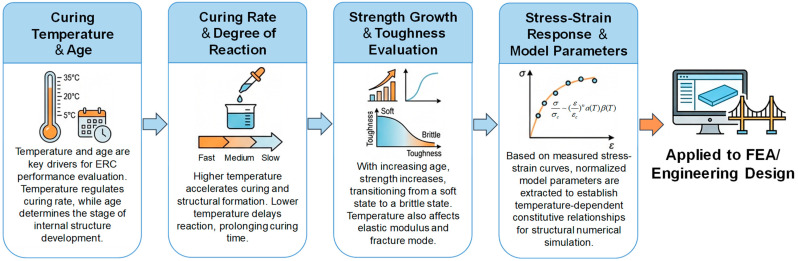
Mechanistic diagram of ERC performance evolution and modeling under different curing temperatures and ages.

To visually illustrate the entire process of ERC evolution—from structure development under environmental factors to constitutive modeling—Figure 8 presents a mechanistic diagram. This diagram outlines a complete pathway from temperature and age inputs to mechanical performance outputs, encompassing key stages such as curing rate, strain capacity, strength evolution, and parameter extraction. This framework provides a theoretical basis for model development and engineering application.

### 5.2. Mechanical Properties

ERC relies on epoxy resin as its primary binding phase, with its mechanical performance fundamentally dependent on the bonding effectiveness of the resin matrix. This research shows that both compressive and tensile strengths increase continuously with curing age. The compressive strength development law is consistent with the research results of J. Shi et al. [33]. The study [33] further indicates that the most rapid strength gain occurs within the early stage (2 h to 1 d), with the 1-day compressive strength exceeding 60% of the 28-day value. In this paper, the 1-day compressive strength exceeded 13 MPa across all temperature conditions, representing 23.87%, 25.09%, and 36.31% of the corresponding 28-day strength, respectively. This reflects a characteristic pattern of “rapid early strength development followed by gradual stabilization after 1 day”, which meets the requirements for emergency repair materials. Curing temperature significantly influences the mechanical performance of ERC. Peak stress is slightly higher in the 20–35 °C range compared to that at 5 °C, indicating that moderate heating enhances load-bearing capacity.

The compressive elastic modulus of ERC rises rapidly with age, increasing from approximately 300 MPa at early ages to 15.7 GPa at 90 days—an increase of over 50 times. After 7 days, the growth rate of the elastic modulus accelerates significantly, reflecting the stiffness leap characteristic of the material during the structural hardening phase in the mid-to-long term.

### 5.3. Constitutive Model

C. Vipulanandan et al. [34] proposed a model considering the effects of strength, temperature and strain rate to predict the compressive stress–strain behavior of polymer concrete. The stress–strain curve can be described by:

(9) $$\frac{\sigma }{\sigma_{c}}=\frac{\left(\alpha_{1}+\alpha_{2}T+\alpha_{3}\log\overset{\cdot }{\varepsilon })(\frac{\varepsilon }{\varepsilon_{c}}\right)}{(\alpha_{1}+\alpha_{2}T+\alpha_{3}\log\overset{\cdot }{\varepsilon }-1)+{\left(\frac{\varepsilon }{\varepsilon_{c}}\right)}^{\left(\alpha_{1}+\alpha_{2}T+\alpha_{3}\log\overset{\cdot }{\varepsilon }\right)}}$$

where *T* is the temperature (°C), $\overset{\cdot }{\varepsilon }$ is the strain rate (min^−1^), and α_1_, α_2_ (/°C), and α_3_ (min) are materials constants. with determined values of 3.179, 0.075 and 0.476, respectively. *σ* represents stress and *ε* represents strain. *σ_c_* is the peak stress and *ε_c_* is the peak strain.

**Figure 9:**
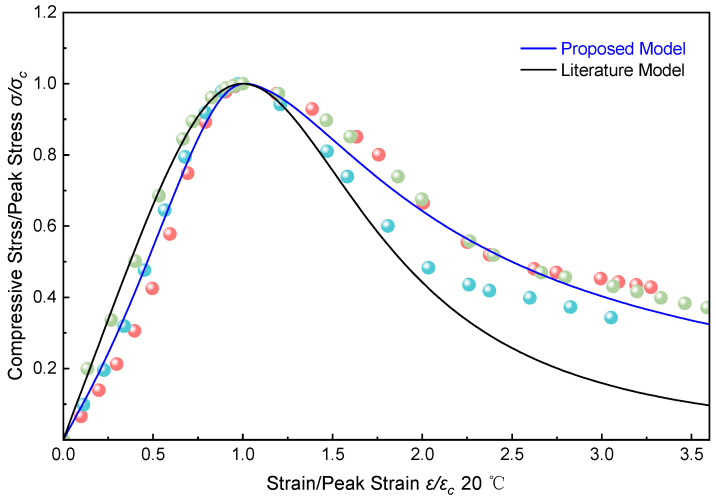
Comparison of the proposed model with the model from the literature [34].

Taking the compressive test data at *T* = 20 °C and a strain rate of $\overset{\cdot }{\varepsilon }$ = 2%/min as an example, the proposed model was compared with the model developed by C. Vipulanandan et al. [34]. As shown in Figure 9, both theoretical curves exhibit excellent continuity and overall smoothness without significant fluctuations, indicating that both models are suitable for continuous numerical simulations. The three-color data points represent the three sets of compression test data.

Ascending Branch: In the initial stage (ε/ε_c_ < 0.5), the proposed model follows the experimental data, accurately capturing the elastic stress–strain response and reliably reflecting the material’s elastic modulus. However, in the region 0.5 < ε/ε_c_ < 1.0, slight deviations from some experimental data points are observed. In contrast, the constitutive model by C. Vipulanandan et al. [34] significantly overestimates the experimental data in the ε/ε_c_ < 0.5 stage, demonstrating limited accuracy in describing the low-strain response. Nevertheless, it exhibits smaller deviations in the 0.5 < ε/ε_c_ < 1.0 range and reasonably represents the actual stress–strain response during this phase.

Descending Branch: The proposed model captures the post-peak softening behavior well, with a descending trend consistent with the experimental data. In contrast, the model by Vipulanandan et al. [34] exhibits a considerably steeper drop after the peak and fails to accurately represent the material’s actual softening response. Furthermore, in the high-strain region (ε/ε_c_ > 2.5), this model displays excessive softening prediction.

The comparative evaluation confirms that the proposed model achieves closer agreement with the experimental data and accurately captures both the peak strength and the hardening behavior of the ERC material.

In fitting the relationship between temperature and shape parameters, the proposed constitutive model achieved a coefficient of determination (*R*^2^) of 1.0. This can be attributed to two factors. First, the model is grounded in a well-established theoretical framework for concrete constitutive behavior. The near-perfect fit indicates that it captures the dominant mechanical response of ERC, rather than merely fitting numerical noise. Second, the fitting procedure was not applied directly to raw experimental data, but to the calibrated shape parameters *α* and *β*, which inherently results in an excellent match.

Although the fitted curves demonstrate outstanding agreement with the experimental data, the generalizability of the proposed model requires further validation using independent datasets, such as those involving different mix proportions, loading regimes, or curing temperatures. Such validation is essential to confirm that the high *R*^2^ values reflect genuine predictive capability rather than overfitting to a specific dataset.

## 6. Conclusions

This study developed a high-performance epoxy resin concrete (ERC) material and systematically investigated the effects of curing ages on its mechanical properties. Furthermore, a temperature-dependent constitutive model was established for engineering applications. The main conclusions are as follows:

(1) An optimized mix proportion was determined: 450 kg of epoxy resin, 74 kg of curing agent, and 1950 kg of graded crushed stone per cubic meter of material. Under typical on-site temperatures (5 °C to 35 °C), ERC cures within 2–6 h and achieves compressive strength exceeding 15 MPa at 1 day, meeting rapid-repair requirements.
(2) ERC exhibits stable, continuous strength development with age, reaching a compressive strength of over 60 MPa, a tensile strength of 10 MPa, and a compressive elastic modulus of approximately 16 GPa after 90 days.
(3) High-temperature curing significantly accelerates the early strength development of ERC, yet its influence on long-term strength remains limited, with only approximately a 10% increase compared to room-temperature curing. In contrast, low-temperature curing not only delays early strength gain but also reduces the ultimate strength by 10–20% relative to that achieved under room-temperature curing.
(4) A temperature-sensitive constitutive model was established based on uniaxial stress–strain data, providing a unified description of ERC behavior under varying thermal conditions. All fitted *R*^2^ values exceed 0.9392, confirming the model’s accuracy in representing the stress–strain relationship.

In summary, the proposed ERC exhibits significant advantages in rapid curing, early strength development, and temperature adaptability. The established temperature-dependent constitutive model effectively captures its mechanical response characteristics. These results provide theoretical and technical support for the rapid repair of bridge expansion joints and enable reliable numerical simulation of ERC-based structures.

Nevertheless, future work will include validation against independent data to further assess the model’s robustness under broader operational scenarios. Subsequent research should also investigate the evolution of mechanical properties and constitutive behavior under the combined effects of multiple influencing factors, including temperature, humidity, and fatigue loading.

## Figures and Tables

**Table 1:** Specimen dimensions and testing standards for ERC.

Type	Shape	Dimensions (mm)	Specimens per Group	Variables	Total Number	Reference Standard
Compression Tests	Cube	100 × 100 × 100	3	Temperature (3 levels) × Curing age (9 levels)	81	British Standard [21]
Tension Test	Shape-8	25 × 25 × 25	3	Temperature (3 levels) × Curing age (9 levels)	90	British Standard [21]
Elastic Modulus	Prism	40 × 40 × 160	6	Curing age (6 levels)	36	JTG E30-2005 [22]

**Table 2:** Failure modes of ERC under compression.

Age	Low-Temperature 5 °C	Room Temperature 20 °C	High-Temperature 35 °C
1 d	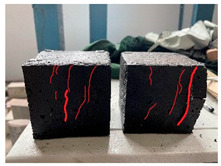	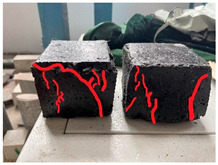	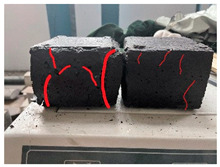
2 d	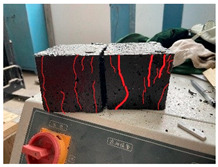	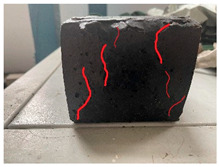	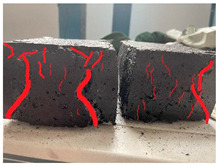
3 d	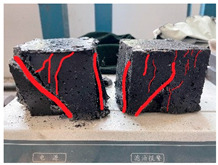	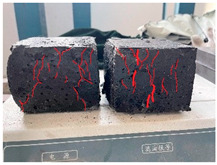	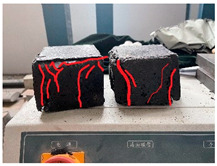
7 d	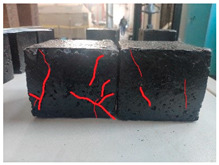	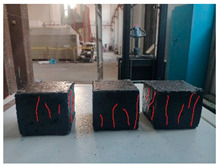	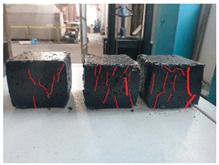
28 d	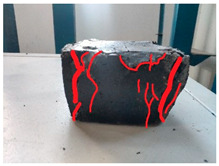	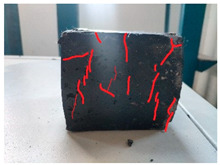	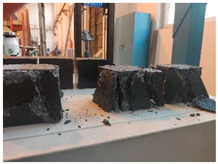
90 d	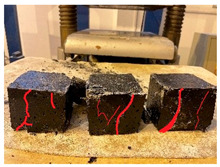	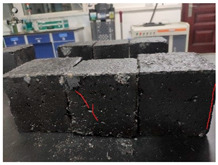	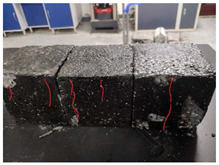

**Table 3:** Failure modes of ERC materials under tension.

Age	Low-Temperature 5 °C	Room Temperature 20 °C	High-Temperature 35 °C
1 d	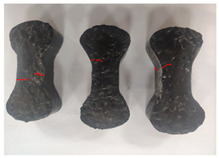	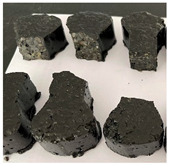	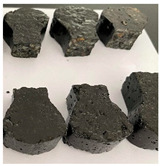
3 d	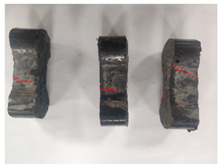	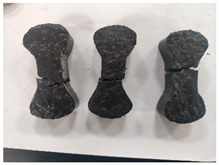	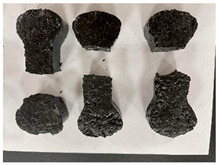
7 d	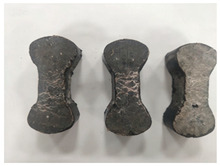	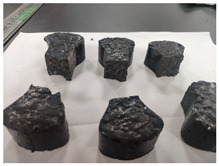	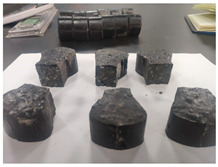
28 d	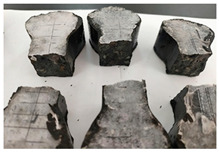	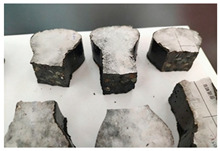	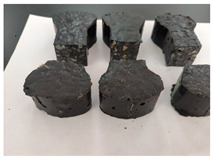
90 d	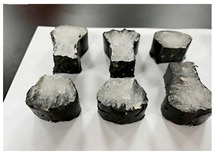	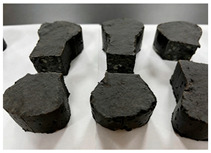	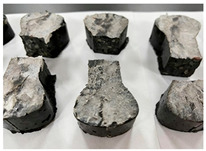

**Table 4:** Fitting coefficients for the compressive constitutive model of ERC.

Parameter	Value	*R* ^2^
*A*_1_	0.06649	1.0
*B*_1_	0.06504	
*C*_1_	−0.00115	
*A*_2_	5.84136	1.0
*B*_2_	−0.45991	
*C*_2_	0.01117	

**Table 5:** Fitting coefficients for the tensile constitutive model of ERC.

Parameter	Value	*R* ^2^
*A*	0.68876	1.0
*B*	−0.01742	
*C*	0.00048	

## Data Availability

The original contributions presented in this study are included in the article. Further inquiries can be directed to the corresponding author.
